# Lower extremity amputation rates in people with diabetes as an indicator of health systems performance. A critical appraisal of the data collection 2000–2011 by the Organization for Economic Cooperation and Development (OECD)

**DOI:** 10.1007/s00592-016-0879-4

**Published:** 2016-07-21

**Authors:** F. Carinci, M. Massi Benedetti, N. S. Klazinga, L. Uccioli

**Affiliations:** 1 Professor of Health Systems and Policy, School of Health Sciences, Faculty of Health and Medical Sciences, Duke of Kent Building, University of Surrey, Guildford Surrey, GU2 7XH UK; 2 Hub for International Health Research, Perugia, Italy; 3 Organization for Economic Cooperation and Development (OECD), Paris, France; 4 Academic Medical Centre, University of Amsterdam, Amsterdam, The Netherlands; 5 Università Tor Vergata, Rome, Italy

**Keywords:** Lower extremity amputation, Diabetes, OECD, Health care quality indicators, Health systems performance, International comparisons

## Abstract

**Aims:**

Critical appraisal of secondary data made available by the OECD for the time frame 2000–2011.

**Methods:**

Comparison of trends and variation of amputations in people with diabetes across OECD countries. Generalized estimating equations to test the statistical significance of the annual change adjusting for major potential confounders.

**Results:**

A total of 26 OECD countries contributed to the OECD data collection for at least 1 year in the reference time frame, showing a decline in rates of over 40 %, from a mean of 13.2 (median 9.4, range 5.1–28.1) to 7.8 amputations per 100,000 in the general population (9.9, 1.0–18.4). The multivariate model showed an average decrease equal to −0.27 per 100,000 per year (*p* = 0.015), adjusted by structural characteristics of health systems, showing lower amputation rates for health systems financed by public taxation (−4.55 per 100,000 compared to insurance based, *p* = 0.002) and non-ICD coding mechanisms (−7.04 per 100,000 compared to ICD-derived, *p* = 0.001). Twelve-year decrease was stronger among insurance-based financing systems (tax based: −0.16 per 100,000, *p* = 0.064; insurance based: −0.36 per 100,000; *p* = 0.046).

**Conclusions:**

In OECD countries, amputation rates in diabetes continuously decreased over 12 years. Still, in 2011, one amputation every 7 min could be directly attributed to diabetes. Although interesting, these results should be taken with extreme caution, until common definitions are improved and data quality issues, e.g., a different ability in capturing diabetes diagnoses, are fully resolved.

## Introduction

Although recognized as an essential target in the clinical management of a person with diabetes, use of lower extremity amputations at system level appears still limited and highly controversial [1].

In 1989, the St.Vincent Declaration launched by WHO Europe and IDF Europe triggered the attention of governments on “reducing by one half the rate of limb amputations for diabetic gangrene” [2]. After over 25 years, there is still insufficient information available at the international level to monitor progress in this direction [3].

The relatively few national audits routinely reporting lower extremity amputations rates in diabetes (LEARD) show a consistent reduction over the years, with a steady state reached in several cases [4–11].

Since 2001, the Organization for Economic Cooperation and Development (OECD) has been collecting a series of indicators as a core activity of the “Health Care Quality Indicators” (HCQI) Project [12], with the aim of developing and reporting international comparisons on the various dimensions of quality of care within a broader conceptual framework of health systems performance [13].

Data collection on LEARD started in 2006 as one of the indicators included in the “primary care” theme of the HCQI. However, annual trends were never published in “Health at a Glance,” the biannual flagship HCQI publication targeted at the broad audience [14].

The main reason for not publishing the international trends of LEARD was a general disagreement among countries on how to cope with the observed variability in the results, how to consider the different coding mechanisms and data sources used to apply common definitions, and which methods would be required for statistical analysis. Consequently, the availability of these data has been substantially underexploited. Meanwhile, LEARD data collected by the OECD have been regularly uploaded to the official OECD online repository StatExtracts (http://stats.oecd.org/).

In the present paper, we present the results of a critical appraisal of the LEARD data collected by the OECD for the years 2000–2011, with the aim of responding to the following main questions:

- How was the trend over 12 years?
- How did rates vary across OECD countries in each year?
- Can amputation rates be used to evaluate quality of care and health systems performance?
- How to enhance international comparability?

## Materials and methods

Standardized LEARD for the time frame 2000–2011 were downloaded from StatExtracts in May 2015, with data referred to the HCQI data collection 2013.

The entire time series was computed by the OECD, using numerators/denominators stratified by sex and age classes, delivered by countries on the basis of the following agreed criteria:

- Numerators include all non-maternal/non-neonatal hospital admissions of subjects aged 15 or over with procedure code of lower extremity amputation (excluding toe) and diagnosis code of diabetes in any field in a specified year. Specific procedure codes (84.10, 84.12–19) were provided only for ICD-9-CM and ICD-10-WHO. In other cases, countries were asked to identify the most appropriate codes for their national classification systems.
- Cases are excluded from the numerators if they are transferred from another institution or present a trauma diagnosis, or same day/day only admissions.
- Denominators refer to the total population aged 15 years or over.
- All rates are expressed as the total number of amputations ×100,000 total general population, standardized (SR) using the total OECD population across 34 member states (MS) in year 2005.

Additional data extracted from the OECD online repository included structural characteristics of data (use of registries and coding systems) and health systems of participating countries (tax-based systems vs insurance based) [15].

Since the USA included amputation of toes in the numerator before 2010, all data reported prior to that date were excluded from the analysis.

Descriptive measures included: measures of centrality (mean, median and range); dispersion (coefficient of variation x 100: CV), total general population aged 15 and over in all 34 MS; and projected total number of amputations per year (computed as a product between the total population and the relative standardized rate). A graphical plot superimposing boxplots to turnip charts and a continuous line representing the average trend for the entire pool of OECD countries, was used to display results over time [16].

Multivariate linear regression using generalized estimating equations (GEE) was used to estimate the average change of standardized LEARD over time, taking into account all structural characteristics identified above as potential confounders and including countries as clusters, with an exchangeable correlation matrix of rates over time. The adoption of GEE models was justified by the need of ensuring robust confidence intervals when the normality assumption is violated by correlated values within clusters (in this case, countries) and missing data are sparsely present [17].

Sensitivity analysis was carried out to take into account the different composition of countries over time, testing the heterogeneity of results obtained for the whole period against those excluding observations prior to 2006, when a limited set of countries was included in the database. The GEE model allowed using all information available at each point in time, rather than just the annual average.

All analyses were performed using the R statistical language [18].

## Results

An expanded version of the OECD data on LEARD for the time frame 2000–2011 is presented in Table 1 reporting standardized rates for each contributing country. Descriptive measures and the projected total number of amputations in the whole OECD area are presented in Table 2.

**Table 1:** Lower extremity amputation rates in diabetes: age-sex standardized rates per 100,000 population aged 15 or over, according to OECD definitions, 2000–2011. *Source* OECD health care quality indicators project (revised version, data collection 2013)

OECD Country	T	R	C	2000	2001	2002	2003	2004	2005	2006	2007	2008	2009	2010	2011
Australia	**Y**	N	2									6.9	6.4	5.5	5.0
Belgium	N	N	1	17.5	18.5	20.0	19.9	20.6	20.6	22.1	20.1	15.9	15.9		
Canada	**Y**	N	2								11.9		10.0	9.6	10.0
Denmark	**Y**	**Y**	2								22.3		19.2		
Finland	**Y**	**Y**	**3**								6.8	6.4	6.5	6.5	5.8
France	N	N	**3**								13.4			7.4	7.1
Germany	N	N	2								36.4		20.6		18.4
Hungary	N	N	2					0.6	0.7	0.8	1.1	1.5	0.7	1.0	1.1
Iceland	**Y**	**Y**	**3**	5.7	3.1	2.5	4.6	2.8	1.1	1.6	0.0	0.4	0.0		
Ireland	**Y**	N	2	5.1	4.8	3.6	5.2	4.3	5.3	4.4	5.6	5.0	4.9	5.2	3.8
Israel	N	N	1	28.1	27.3	26.8	27.4	26.2	26.3	25.7	25.1	20.7	18.6	19.5	
Italy	**Y**	N	1		5.9	6.7	6.6	6.4	6.4	5.8	5.6	5.9	5.7	5.9	5.7
Korea	N	N	**3**								8.8	9.4	9.6	9.5	9.8
Luxembourg	N	N	**3**			4.9	7.7	4.8	5.5	3.6	7.7	6.0	4.6	7.0	2.8
Mexico	N	N	2										8.3	9.5	9.2
Netherlands	N	**Y**	**3**						12.5		12.8	11.6	11.6	13.5	
New Zealand	**Y**	N	2						8.5	6.7	7.0	7.9	7.6	7.1	6.7
Norway	**Y**	**Y**	**3**								11		9.3	7.8	8.7
Poland	N	N	2						14.0	15.3	12.6	12.8	13.3	13.9	
Portugal	**Y**	N	1												12.8
Slovenia	N	N	2										14.0	12.5	15.1
Spain	**Y**	N	1	9.4	9.9	10.1	10.6	10.5	10.5	10.8	10.5	10.4	10.6	9.7	9.6
Sweden	**Y**	**Y**	**3**								3.2	3.2	3.6	3.2	3.3
Switzerland	N	**Y**	1							16.8		7.9		7.1	
United Kingdom	**Y**	N	**3**							5.2	5.3	5.4	5.3	5.3	5.1
United States	N	N	1											17.1	

*T* funding mechanism = tax based (Y/N), *R* source = registry (Y/N), *C* coding = ICD-9 derived (1), ICD-10 derived (2), or Other (3); Codes in *bold* denote categories used in the multivariate model vs reference (union of codes in flat text). *Age Group* 15 years old and over; Austria, Chile, Czech Republic, Estonia, Greece, Japan, Slovak Republic, Turkey (all insurance based) did not contribute to the data collection

**Table 2:** Lower extremity amputation rates in diabetes: descriptive measures according to OECD definitions, 2000–2011. *Source* OECD health care quality indicators project (revised version, data collection 2013)

*N* contributing countries	5	6	7	7	8	11	13	20	18	22	21	18
Mean SR	13.2	11.6	10.7	11.7	9.5	10.1	9.9	11.4	9.08	9.4	8.8	7.8
Median SR	9.4	7.9	6.7	7.7	5.6	8.5	6.2	9.6	6.9	8.8	7.4	6.9
Range SR	5.1–28.1	3.1–27.3	2.5–26.8	4.6–27.4	0.6–26.2	0.7–26.3	0.8–25.7	0–36.4	0.4–20.7	0–26.6	1–19.5	1.0–18.4
CV	73.7	81.7	86.8	73.9	96.0	78.0	83.2	77.8	63.3	60.9	51.2	57.2
Total OECD population 15+	914,500,500	923,611,800	932,567,800	941,187,300	949,122,600	960,511,700	969,778,100	979,293,900	989,353,400	996,893,200	1,005,814,200	1,014,983,900
Overall no. amputations^a^	120,348	106,985	99,385	110,253	90,404	97,274	116,597	111,248	94,428	93,481	88,033	78,943

^a^Estimated among people with diabetes aged 15+ in OECD countries

A total of 26 OECD countries contributed to the HCQI data collection on LEARD with at least one year in the reference time frame. One half of MS presents only data from 2006 onwards. A total of 8 MS did not participate and were only considered for the total population used for standardization: Austria, Chile, Czech Republic, Estonia, Greece, Japan, Slovak Republic and Turkey.

Overall, standardized LEARD experienced a 12-year decline of over 40 %, from a mean of 13.2 amputations per 100,000 in 2000 (median, range: 9.4, 5.1–28.1) to a mean of 7.8 amputations per 100,000 in 2011 (9.9, 1.0–18.4).

**Fig. 1:**
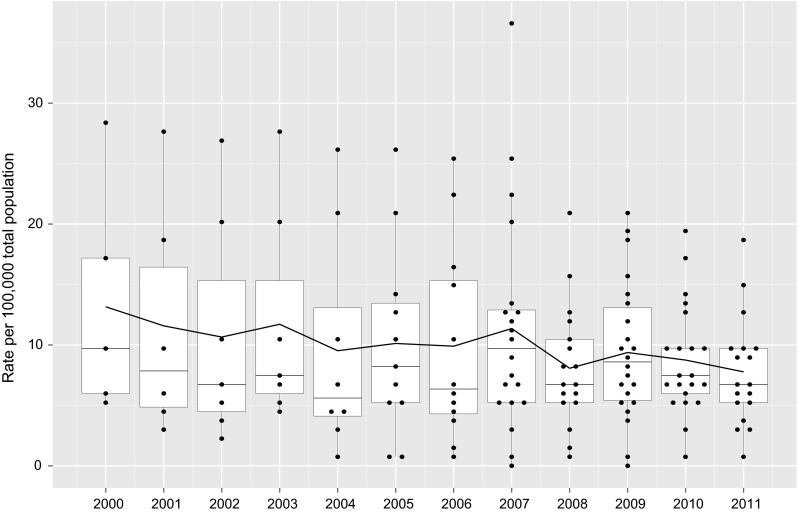
Lower extremity amputation rates in diabetes, OECD 2000−2011

The average reduction appeared to be consistently linear over time (Fig. 1), with the exception of 2007, a year in which Germany entered the time series with a very high value.

The coefficient of variation is very high across the whole period, although declining fairly linearly from 73.7 % in 2000 to 57.2 % in 2011. In this year, we still find values as high as 18.4 per 100,000 for Germany, as opposed to its near neighbor country, Hungary, which reports a very low value of 1.1 per 100,000.

The results produced using only rates from 2006 onwards did not substantially differ from those relative to the whole time frame. Therefore, the GEE outputs presented in Table 3 refer to all information available between 2000 and 2011.

**Table 3:** Results of multivariate linear regression (generalized estimating equations), OECD 2000–2011 *Source* OECD health system characteristics survey, 2012; health care quality indicators project (revised version, data collection 2013)

Model/Variable	Estimate	S.E.	95 %C.I.	*P* > *Z*
Model 1 [Complete dataset; N countries = 26]				
Tax-based system	−4.55	1.95	−8.38, −0.72	0.020
Use of registry	2.93	2.53	−2.03, 7.89	0.247
Non-ICD coding	−7.04	2.14	−11.24, −2.84	0.001
Average year change	−0.27	0.11	−0.50, −0.05	0.015
Model 2 [Financing: Tax-based; N countries = 12; Median LEARD: 7.55 (2000), 6.25 (2011)]				
Average Year Change	−0.16	0.09	−0.33, 0.01	0.064
Model 2 [Financing: Social insurance; N countries = 14; Median LEARD: 17.50 (2000), 8.15 (2011)]				
Average year change	−0.36	0.18	−0.71, −0.01	0.046

The first model shows that LEARD have significantly decreased on average by −0.27 per 100,000 per year (95 % CI: −0.50, −0.05; *p* = 0.015), equal to −3.30 per 100,000 over 12 years, adjusted by financing mechanism, use of registry and coding system.

Countries with a tax-based financing system present a significant difference in standardized LEARD equal to −4.55 per 100,000 (95 %CI: −8.38, −0.72; *p* = 0.002). Using a registry as a data source, albeit not significant (+2.9 per 100,000, 95 %CI: −2.03, 7.8925; *p* = 0.247), was retained into the model to ensure adjustment for a potential confounder.

No significant difference was found between countries using ICD-9 vs ICD-10. Thus, the two categories were merged in a reference category against non-ICD classification systems, the latter presenting significantly lower amputation rates equal to −7.04 per 100,000 (95 %CI: −11.24, −2.84; *p* = 0.001).

The GEE models separately run on tax-based vs insurance-based health systems revealed that the reduction in LEARD was not homogeneous among groups. Among tax-based systems (Model 2), the annual decrease was low and not significant (−0.16, *p* = 0.064), while insurance-based health systems (Model 3) showed a statistically significant, stronger than average decrease equal to −0.36 per 100,000 (95 %CI: −0.71, −0.01; *p* = 0.046).

Notably, tax-based countries started from a much lower average level of LEARD compared to insurance-based (7.55 vs 17.50 per 100,000 in 2000, as opposed to 6.25 vs 8.15 per 100,000 in 2011). These results indicate that the relative performance of countries may depend upon varying characteristics as well as the trend dynamics.

## Discussion

The revised analysis of the OECD data relative to 12 consecutive years suggests relevant answers to our initial set of questions. The confirmed links between lower limb amputations, disability and excess mortality [19–21] call for immediate action on a global scale. In 2011, across the whole OECD area, over 216 amputations per day could be directly attributed to diabetes.

The variability between countries is still relevant and in certain cases difficult to explain: Germany has a rate of over 18 times higher than Hungary (18.4 vs 1.1 per 100,000). Imbalances in the prevalence of diabetes may represent a potential primary reason, but there might be also other possible explanations, involving different types of factors.

For instance, the result for Germany may be biased by a higher number of minor amputations in the numerator or lack of accurate data, given that there is no national register to verify the precision of these estimates. At the same time, a recent publication highlighted a lack of guideline adherence in managing peripheral arterial disease and critical limb ischemia [22].

On the other hand, data reported for Hungary seem particularly unclear, with rates remaining fairly stable at minimal levels since 2004. The fact that other countries with long-standing tradition in diabetes foot care present significantly higher values may question the comparability of these national results.

Across the whole time frame, there has been a statistical significant reduction in amputation rates in diabetes, indicating a potential beneficial effect of guidelines and policies implemented by all countries to enhance the quality of care for diabetes patients. Adjusting by the main potential confounders, the overall reduction in standardized rates over 12 years was equal to −3.30 per 100,000, slightly lower than the difference by over 40 % between the average rates of 2000–2011, which can be interpreted as a remarkable success.

Projected over the entire OECD total general population, the overall reduction in average standardized rates translates into an estimated decrease in the absolute number of amputations in diabetes corresponding to 41,405 per year, from 120,348 in 2000 to 78,943 in 2011. Such a decrease is also accompanied by a similar trend toward lower variation between countries, as shown by decreasing coefficients of variation. These results appear particularly positive, considering the general increase in the prevalence of diabetes experienced worldwide during the same time interval [23].

The results appear more controversial when comparing health systems with different funding mechanisms. The average difference in favor of tax-based vs insurance-based systems is equal to 4.5 per 100,000 diabetic amputations. On the other hand, the improvement has been stronger for insurance-based systems, corresponding to a significant decrease of −0.36 per 100,000 per year. This can be largely explained by the fact that the starting point was much higher for that group of countries. In fact, the two financing groups correspond to high and low performers in an alternate way, depending on whether one considers a point in the time series or the average improvement over time.

The above results seem to highlight recent successes as well as enduring challenges in the planned reduction in amputation rates in people with diabetes. Although interesting, they should be also taken with extreme caution.

In the OECD data collection, countries are fully in charge of ensuring accurate reporting of cases of diabetes. Therefore, we could not assess to what extent a systematic underreporting of diabetes diagnoses may have determined lower rates in specific cases. Until data quality issues such as this one are not fully resolved, the level of association found between insurance-based systems and higher rates should be only used to raise hypotheses for further investigation, but by no means can be intended as a cause–effect relationship.

On the other hand, the significantly lower rates found for countries using non-ICD classification systems should not be interpreted in relation to data quality. In fact, systems, e.g., NOMESCO, are prevalently used in countries (e.g., the Nordic) that apply data linkage more extensively, use more quality registries and thus are more likely to report accurate numerators (as indirectly confirmed by the coefficient of registry use in the multivariate model). Potential bias may have been induced by the inclusion of specifications only related to ICD systems in the OECD guidelines. The inclusion of coding as an adjustment term in the multivariate model provided us with a more balanced estimate of the average annual reduction in amputations.

The inclusion of additional potential confounders, e.g., diabetes prevalence, was discarded for specific reasons. Firstly, because stratified estimates of disease-oriented measures, e.g., diabetes prevalence, are still difficult to obtain from national governments, while our approach is based only on official data. Secondly, because it would be preferable not to adjust for factors that could be directly associated to health systems performance, a point of major interest in the present study. As a matter of fact, diabetes-related factors were embedded in the overall measurement of quality of care provided by LEARD in the general population (consistently with the denominator).

Specific methodological recommendations can be suggested for future data collection:

- Further efforts must be made to embed in the data collection means to ascertain (and possibly reduce) any cause of systematic underreporting of diabetes diagnoses (as in the case of Hungary).
- Data are still of insufficient granularity to draw conclusions on quality of care.
  - How to interpret the reported reduction when considering major vs minor amputations? Minor amputations may indicate better quality of care as an intervention to prevent major ones and hence salvage lower extremities. A stable number of the total number of amputations, or even an increase, may actually hide a higher number of minor vs major, which in turn should be interpreted as a better performance. However, the data collection presented here cannot capture the clinical relevance of minor and major amputations. To this end, the OECD indicator should explicitly distinguish them.
  - Amputation rates calculated over the total general population may hide significant improvements in the quality of care provided to a higher number of subjects with diabetes, as certified by the steep global increase in prevalence. To provide unbiased comparisons between countries, it is essential that the OECD denominator is only referring to the number of subjects with diabetes.
  - Further refinement would require avoiding double counts of amputations and referring to the percentage of subjects with diabetes experiencing major vs minor amputations. In this case, it would be preferable to monitor patients over time through a unique patient identifier, considering only the most severe amputation within a year to classify an amputee in either the “minor” or the “major” category at each term.

The above improvements to indicator reporting would allow testing the validity of our results in more detail, confirming the existence of significant differences between countries that can lead to active interventions.

International organizations, e.g., the OECD, should specifically use this knowledge to influence the identification of effective policies for quality of care improvement.

In particular, the observed advantage of tax-based systems in diabetes care may indicate more complicated underlying processes that deserve to be carefully explored. For instance, tax-based financing systems may represent only a proxy for the different ways of organizing continuous care for diabetes patients and services aimed at reducing complications. This consideration calls for more in depth comparisons between differing types of practices, rather than countries.

Healthcare systems can be differently organized in terms of chronic disease management and integrated care, or may have different availability of specialist centers, unequal access to treatments, e.g., insulin, reagent strips and/or lack of solutions, e.g., specialized centers for the diabetic foot, extensive use of health information systems, broad presence of educational programs and activities for consumers empowerment.

On the other hand, the different ability to track diabetes diagnoses and potential issues in the data quality affecting LEARD estimation may not allow, at this point, to derive definitive conclusions in this direction. More work is required to undertake international comparisons of LEARD.

Our report suggests that the computation of amputation rates has the potential to highlight strengths and weaknesses of health systems in terms of data infrastructure as well as healthcare quality. For this reason, the OECD should continue to retain LEARD as a useful tool in the total set of Health Care Quality Indicators.

The present report suggests that amputation rates may well fit the analysis of quality of care to help understanding which policies work better, rather than producing a league table to rank countries according to their performance.

More refined indicators will require strengthening the information infrastructure, through the implementation of common standards that would assure minimal data quality requirements. This is an effort that may not be as straightforward as it may eventually seem. To use it on a regular basis, governments should agree upon the essential levels of health information required to guarantee international comparability and adequate support for quality improvement strategies [24].

We encourage the OECD to continue along this process and further refine the definitions and data collection of LEARD, in ways that could shed light on the result of policies implemented at national level.

The preliminary results from the most recent OECD data collection relative to year 2013, published in Health at a Glance 2015, have already made a step ahead in this direction [25].

## Conclusions

Despite an overall significant reduction observed across 12 consecutive years, amputation rates among people with diabetes remain still high in most OECD countries. In 2011, one amputation every 7 min among subjects aged 15 years or over could be directly attributed to diabetes.

Our analysis of OECD data from 26 countries shows lower amputation rates in health systems financed by public taxation, taking into account different coding mechanisms. These results encourage to continue the exploration on whether amputation rates could play a primary role in the quality matrix adopted by the OECD for the general evaluation of health systems, in strict collaboration with national governments [13].

Although interesting, these results should be taken with extreme caution, until common definitions are improved and data quality issues, e.g., a different ability in capturing diabetes diagnoses, are fully resolved.

The debate on how to make the best use of the knowledge acquired on LEARD shall continue at different levels, along with efforts aimed at improving international comparability. Our results provide an avenue for the OECD to continue on this pathway, in direct collaboration with researchers, health professionals and policy makers.

## References

[1] Margolis DJ, Jeffcoate W (2013). Epidemiology of foot ulceration and amputation: can global variation be explained?. Med Clin North Am.

[2] WHO, IDF Europe, Diabetes care and Research in Europe: The St. Vincent declaration, IDF 1989. http://www.codex.vr.se/texts/SVD.pdf. Accessed 4 June 2016

[3] Moxey PW, Gogalniceanu P, Hinchliffe RJ, Loftus IM, Jones KJ, Thompson MM, Holt PJ (2011). Lower extremity amputations—a review of global variability in incidence. Diabet Med.

[4] Gregg EW, Li Y, Wang J, Burrows NR, Ali MK, Rolka D, Williams DE, Geiss L (2014). Changes in diabetes-related complications in the United States, 1990–2010. N Engl J Med.

[5] Lombardo FL, Maggini M, De Bellis A, Seghieri G, Anichini R (2014). Lower extremity amputations in persons with and without diabetes in Italy: 2001–2010. PLoS ONE.

[6] Winell K, Venermo M, Ikonen T, Sund R (2013). Indicators for comparing the incidence of diabetic amputations: a nationwide population-based register study. Eur J Vasc Endovasc Surg.

[7] Holman N, Young RJ, Jeffcoate WJ (2012). Variation in the recorded incidence of amputation of the lower limb in England. Diabetologia.

[8] Kennon B, Leese GP, Cochrane L, Colhoun H, Wild S, Stang D, Sattar N, Pearson D, Lindsay RS, Morris AD, Livingstone S, Young M, McKnight J, Cunningham S (2012). Reduced incidence of lower-extremity amputations in people with diabetes in Scotland: a nationwide study. Diabetes Care.

[9] Buckley CM, O’Farrell A, Canavan RJ, Lynch AD, De La Harpe DV, Bradley CP, Perry IJ (2012). Trends in the incidence of lower extremity amputations in people with and without diabetes over a five-year period in the Republic of Ireland. PLoS ONE.

[10] López-de-Andrés A, Martínez-Huedo MA, Carrasco-Garrido P, Hernández-Barrera V, Gil-de-Miguel A, Jiménez-García R (2011). Trends in lower-extremity amputations in people with and without diabetes in Spain, 2001–2008. Diabetes Care.

[11] Fosse S, Hartemann-Heurtier A, Jacqueminet S, Van Ha G, Grimaldi A, Fagot-Campagna A (2009). Incidence and characteristics of lower limb amputations in people with diabetes. Diabet Med.

[12] Mattke S, Epstein AM, Leatherman, S (2006) The OECD health care quality indicators project: history and background. Int J Qual Health Care, 18 (suppl 1), 1–4, http://intqhc.oxfordjournals.org/content/18/suppl_1/1.full.pdf+html. Accessed 4 June 2016 10.1093/intqhc/mzl019 16954509

[13] Carinci F, Van Gool K, Mainz J et al. (2015) OECD health care quality indicators expert group. Towards actionable international comparisons of health system performance: expert revision of the OECD framework and quality indicators. Int J Qual Health Care.;27:137–146. Epub 2015 Mar 10 http://intqhc.oxfordjournals.org/content/27/2/137.long. Accessed 4 June 2016 10.1093/intqhc/mzv004 25758443

[14] OECD. Health at a Glance 2013: OECD Indicators, OECD Publishing, 2013. http://www.oecd.org/els/health-systems/Health-at-a-Glance-2013.pdf. Accessed 4 June 2016

[15] OECD, The OECD Survey on Health Systems Characteristics, http://www.oecd.org/els/health-systems/characteristics.htm. Accessed 4 June 2016

[16] Carinci F, Di Stanislao F, Moirano F et al. (2014) Italy: Geographic variations in health care, in Geographic variations in health care: what do we know and what can be done to improve health system performance?, OECD http://www.keepeek.com/Digital-Asset-Management/oecd/social-issues-migration-health/geographic-variations-in-health-care_9789264216594-en#page287. Accessed 4 June 2016

[17] Nicolucci A, Carinci F, Graepel J, Hohman T, Ferris R, Lachin JM (1996). The efficacy of tolrestat in the treatment of diabetic peripheral neuropathy: a meta-analysis of individual patient data. Diabetes Care.

[18] R Development Core Team (2005), A language and environment for statistical computing, R foundation for statistical computing, Vienna, Austria, http://www.R-project.org. Accessed 4 June 2016

[19] Hoffmann M, Kujath P, Flemming A, Proß M, Begum N, Zimmermann M, Keck T, Kleemann M, Schloericke E (2015). Survival of diabetes patients with major amputation is comparable to malignant disease. Diabetes Vasc Dis Res.

[20] Hoffstad O, Mitra N, Walsh J et al. (2015) Diabetes, lower-extremity amputation, and death. Diabetes Care; Oct;38(10):1852–1857. http://care.diabetesjournals.org/content/38/10/1852.long. Accessed 4 June 2016 10.2337/dc15-0536 26203063

[21] Giurato L, Vainieri E, Meloni M et al. (2015) Limb salvage in patients with diabetes is not a temporary solution but a life-changing procedure. Diabetes Care published on line August 20, (2015). http://care.diabetesjournals.org/content/38/10/e156.full.pdf. Accessed 4 June 2016 10.2337/dc15-0989 26294664

[22] Reinecke H, Unrath M, Freisinger E, Bunzemeier H, Meyborg M, Lüders F, Gebauer K, Roeder N, Berger K, Malyar NM (2015). Peripheral arterial disease and critical limb ischaemia: still poor outcomes and lack of guideline adherence. Eur Heart J.

[23] Global report on diabetes.World Health Organization 2016. http://www.who.int/diabetes/global-report/en/ Accessed 4 June 2016

[24] Carinci F (2015). Essential levels of health information in Europe: an action plan for a coherent and sustainable infrastructure. Health Policy.

[25] OECD (2015), “Diabetes care”, in Health at a glance 2015: OECD indicators, OECD Publishing, Paris, http://www.oecd-ilibrary.org/social-issues-migration-health/health-at-a-glance-2015/diabetes-care_health_glance-2015-45-en. Accessed 4 June 2016

